# The Immediate Suture of Divided Nerves

**Published:** 1884-02

**Authors:** 


### The Immediate Suture of Divided Nerves.

In the *Glasgow Med. Journal*, October, 1883, Mr. Clark discusses this subject, and concludes that it is eminently proper to at once suture divided nerves. It should be as much a rule of practice to bring together the cut ends of a divided nerve as to stitch the wound in the muscles or skin.

